# Knowing the scope of meningococcal disease in Latin America

**DOI:** 10.26633/RPSP.2017.118

**Published:** 2017-12-20

**Authors:** Marco A. P. Sáfadi, María Teresa Valenzuela, Ana Flavia Carvalho, Lúcia Helena De Oliveira, David M Salisbury, Jon Kim Andrus

**Affiliations:** 1 Santa Casa de São Paulo School of Medical egy to prevent meningococcal disease Santa Casa de São Paulo School of Medical egy to prevent meningococcal disease Sciences São Paulo Brazil Santa Casa de São Paulo School of Medical egy to prevent meningococcal disease. Sciences, São Paulo, Brazil.; 2 Facultad de Medicina Universidad de los Andes Santiago Chile Facultad de Medicina, Universidad de los Andes, Santiago, Chile.; 3 Sabin Vaccine Institute Sabin Vaccine Institute Washington, D.C. United States of America Sabin Vaccine Institute, Washington, D.C., United States of America.; 4 Pan American Health Organization Pan American Health Organization Washington, D.C United States of America Pan American Health Organization, Washington, D.C., United States of America.; 5 Centre on Global Health Security at Chatham House Centre on Global Health Security at Chatham House London United Kingdom Centre on Global Health Security at Chatham House, London, United Kingdom.

**Keywords:** *Neisseria meningitidis*, meningococcal infections, meningitis, vaccines, public health surveillance, Latin America, *Neisseria meningitidis*, infecciones meningocócicas, meningitis, vacunas, vigilancia en salud pública, América Latina, *Neisseria meningitidis*, infecções meningocócicas, meningite, vacinas, vigilância em saúde pública, América Latina

## Abstract

Opportunities for strengthening surveillance of meningococcal disease exist between and within countries in Latin America. In August of 2015, a workshop was convened in the city of São Paulo, Brazil, to address the following objectives: 1) to review meningococcal disease burden and vaccine use in Latin America; 2) to evaluate the effectiveness of current meningococcal surveillance practices in the region; 3) to identify challenges to meningococcal surveillance in the region; and 4) to outline steps for strengthening meningococcal surveillance and disease control in the region. Based on the workshop’s discussions, recommendations for strengthening surveillance and controlling meningococcal disease in Latin America focus on improving: a) laboratory capabilities for diagnostic testing; b) communication regarding epidemiologic- and laboratory-based analyses; c) communication during outbreaks; d) monitoring of long-term disease outcomes; e) knowledge of vaccines against serogroup B disease; and f) criteria for defining and controlling meningococcal outbreaks. Overall, improving surveillance will help guide strategies for meningococcal disease prevention and control in Latin America.

## RESUMEN

Existen distintas oportunidades para reforzar los procedimientos de vigilancia de la enfermedad meningocócica entre los países de América Latina y dentro de ellos. En agosto del 2015, se llevó a cabo un taller en la ciudad de São Paulo (Brasil) en el que se trataron los siguientes puntos: 1) examen de la carga de la enfermedad meningocócica y el uso de la vacuna en América Latina; 2) evaluación de la eficacia de los actuales procedimientos de vigilancia de la enfermedad meningocócica en la región; 3) especificación de los retos para la vigilancia de la enfermedad meningocócica en la región; y 4) definición de los pasos para fortalecer los procedimientos de vigilancia de la enfermedad meningocócica y el control de esta enfermedad en la región. Como resultado del taller se formularon recomendaciones para reforzar los procedimientos de vigilancia y control de la enfermedad meningocócica en América Latina que hacían hincapié en mejorar: a) las capacidades de laboratorio con respecto a las pruebas diagnósticas; b) la comunicación sobre los análisis epidemiológicos y de laboratorio; c) la comunicación durante los brotes; d) el seguimiento de las consecuencias de la enfermedad a largo plazo; e) el conocimiento sobre las vacunas contra la enfermedad causada por el serogrupo B; y f) los criterios para definir y controlar los brotes meningocócicos. En términos generales, la mejora de los procedimientos de vigilancia ayudará a delinear las estrategias para la prevención y el control de la enfermedad meningocócica en América Latina.

## RESUMO

Existem oportunidades para o reforço da vigilância da doença meningocócica entre os países e em cada país na América Latina. Em agosto de 2015, foi realizado um seminário na cidade de São Paulo, Brasil, com os seguintes objetivos: 1) avaliar a carga da doença meningocócica e o uso da vacina na América Latina; 2) avaliar a eficácia das atuais práticas de vigilância da doença meningocócica na Região; 3) identificar os desafios para a vigilância meningocócica na Região e 4) definir medidas para reforçar a vigilância da doença meningocócica e o controle da doença na Região. Partindo dos debates realizados durante o seminário, foram feitas as seguintes recomendações para reforçar a vigilância e o controle da doença meningocócica na América Latina, dando-se ênfase a melhorar: a) a infraestrutura laboratorial para exames diagnósticos; b) a comunicação das análises epidemiológicas e laboratoriais; c) a comunicação nos surtos; d) o monitoramento dos desfechos da doença a longo prazo; e) o conhecimento sobre as vacinas contra o meningococo do sorogrupo B e f) os critérios para definir e controlar os surtos de doença meningocócica. Em geral, a melhoria da vigilância contribuirá para orientar as estratégias para prevenção e controle da doença meningocócica na América Latina.

*Neisseria meningitidis* (*N. meningitidis*) causes invasive meningococcal disease, a serious bacterial infection resulting in meningitis, bacteremia, or sepsis (1). Meningitis is the most common clinical presentation of meningococcal infections. Every year, it is estimated that at least 1.2 million people become sick with meningococcal disease worldwide and 135 000 individuals die (2). Up to 20% of survivors suffer permanent damage, including deafness, brain injuries, and amputated limbs (3). The case-fatality rate ranges from 10% to 15%, is highest among adolescents and adults, and reaches up to 40% in patients with meningococcemia (4). In Latin America, one in five people with meningococcal disease will die (5).

Currently six serogroups—A, B, C, W, Y, and X—are responsible for most cases of invasive meningococcal disease (6). Meningococci have the potential capacity to exchange the genetic material responsible for capsular production and to switch capsular phenotype (7). This capacity and the ongoing evolution of new hypervirulent lineages contribute to the epidemic potential of *N. meningitidis* and call for constant public health surveillance.

Vaccination is the most effective strategy to prevent meningococcal disease. Group A polysaccharide vaccines have demonstrated short-term effectiveness of 85% to 100% in children aged 2 years old and above, as well as in adults (1). Monovalent and multivalent conjugate vaccines are used in immunization programs globally and have reduced the meningococcal disease burden through direct and indirect protection (8). Conjugate vaccines have also demonstrated increased immunogenicity in children less than 2 years of age (1). Due to these benefits, the World Health Organization (WHO) recommends conjugate vaccines over polysaccharide vaccines (1).

Meningococcal vaccines to protect against serogroups A, B, C, W, and Y are currently in use around the world. Widespread use of MenAfriVac, a conjugate vaccine against MenA infection, substantially reduced the incidence of meningitis in countries in the “meningitis belt” of sub-Saharan Africa (2). In the United States of America, the new protein-based MenB vaccines are being used to protect specific high-risk groups. In September 2015, the United Kingdom became the first country to introduce routine vaccination against MenB disease, using Bexsero, in the infant immunization schedule. In Brazil, wide-scale use of MenC vaccine substantially reduced the rate of morbidity and mortality, particularly among those most vulnerable— children under 5 years of age. The dramatic emergence of serogroup W disease in the United Kingdom has prompted the introduction of quadrivalent MenACWY conjugate vaccines in adolescents. In 2012, a meningococcal vaccination program controlled an outbreak of the hypervirulent serogroup W disease in Chile.

On 11–12 August 2015, public health and medical experts from across Latin America met in the city of São Paulo, Brazil, to discuss meningococcal disease surveillance in the region. The workshop focused on the following objectives: 1) to review meningococcal disease burden and vaccine use in Latin America; 2) to evaluate the effectiveness of current meningococcal surveillance practices in the region; 3) to identify challenges to meningococcal surveillance in the region; and 4) to outline steps for strengthening meningococcal surveillance and disease control in the region. This paper reflects the discussions and the conclusions reached during the workshop, focusing on countries’ needs to improve surveillance, lessons learned, and recommendations for the future.

## MENINGOCOCCAL DISEASE BURDEN IN LATIN AMERICA

In most Latin American countries, the true burden of meningococcal disease is underestimated to this day (9). Incidence rates during the last five years differ greatly, ranging from less than 0.1 to 1.8 per 100 000 inhabitants, depending on the country and the year (5). Notably, these rates represent low endemicity (< 2 cases per 100 000) and are below the threshold of “high risk” at which the WHO recommends vaccine use either within a national immunization program or to control outbreaks (1). However, these rates need to be carefully interpreted due to a number of challenges regarding the reporting of meningococcal disease in the region, which may contribute to the differences in incidence.

Across Latin America, serogroup B and serogroup C cause the most disease. Serogroup A has not been circulating for many years. Serogroups B and C particularly affect children under 2 years of age in endemic settings (10). Meanwhile, serogroup W has emerged as a hypervirulent serogroup in Argentina, Brazil, and Chile over the past decade (10). Serogroup Y, which is associated with pneumonia, remains rare in Latin America. Nevertheless, it has been increasing in Argentina, Chile, Colombia, Costa Rica, Uruguay, and Venezuela since 2000 (10).

## MENINGOCOCCAL VACCINE USE IN LATIN AMERICA

A lack of data has contributed to inconsistent policy decisions regarding the use of meningococcal vaccines in Latin America. Among the small number of countries that have made meningococcal vaccines a routine part of their national vaccination programs are Brazil, Chile, Cuba, and, more recently, Argentina. Other nations have used vaccines to curb outbreaks, often in combination with antibiotic prophylaxis. Some countries opt to vaccinate only designated high-risk groups. An ongoing issue involves cost-effectiveness, since in many Latin American countries the disease is relatively rare and the price of meningococcal vaccines is high.

Decision-making for new vaccine introduction requires high-quality epidemiologic data collected through public health surveillance systems. In addition to vaccine safety and efficacy, countries should take into account the burden of disease in the target population for vaccination when considering the introduction of a new vaccine. A country’s disease burden and epidemiologic patterns can only be fully established through national surveillance systems. In turn, post-introduction data may be used to assess the impact of newly implemented vaccines, thus further underscoring the importance for improving surveillance practices.

## MENINGOCOCCAL SURVEILLANCE IN LATIN AMERICA

Surveillance systems monitor the incidence, severity, and serogroup prevalence of disease. After a new vaccine is introduced, surveillance is also essential to monitor the vaccine’s impact on the epidemiologic profile of the disease, carriage, strain distribution, and pathogen behavior.

The surveillance network operated by the Pan American Health Organization (PAHO) is known, in its Spanish acronym, as SIREVA. SIREVA is a passive laboratory-based surveillance network consisting of 21 national reference laboratories and two regional reference laboratories, the Adolfo Lutz Institute in Brazil and the National Health Institute in Colombia.

Participating hospitals voluntarily report laboratory data and some clinical and epidemiological characteristics for cases of invasive *Streptococcus pneumoniae, Haemophilus influenzae,* and *Neisseria meningitidis infection* from children under 5 years of age. These hospitals report data to national ministries of health, and the ministries then report to PAHO. Hospitals also send cerebrospinal fluid (CSF) and blood specimens to national reference laboratories for strain confirmation and for antibiotic susceptibility testing.

In addition to passive surveillance, PAHO has conducted sentinel hospital surveillance of bacterial meningitis and pneumonias since 2005. PAHO recommends that countries implement sentinel surveillance in select hospitals before introducing a meningococcal vaccine, and that they continue the surveillance following vaccine introduction in order to monitor impact and the epidemiological profile of the disease. Currently in Latin America, 13 countries together with 44 hospitals have implemented active sentinel surveillance. Since 2008, PAHO has participated in the WHO-coordinated global Rotavirus and Invasive Bacterial Vaccine Preventable Diseases (IB-VPD) Sentinel Hospital Surveillance Networks (5).

## MENINGOCOCCAL DISEASE CARRIAGE IN LATIN AMERICA

Understanding meningococcal transmission not only requires analyzing active disease infections, but also the strains carried asymptomatically within a population. While most carriers do not become sick, they can transmit the bacteria through respiratory droplets, potentially causing full-blown epidemics. In nonepidemic settings, carriage studies performed around the world revealed that approximately 5% to 10% of the population carry meningococci (5). Adolescents are often carriers.

Chile recently conducted an extensive carriage study after an explosive MenW outbreak with a high case-fatality rate (11). Although the overall prevalence of serogroup W was low, closer examination revealed that within serogroup W there was only one clonal complex, belonging to the highly virulent and invasive ST-11 (12, 13). Tracking disease carriage was critical in showing that hypervirulent clones were circulating in the country, calling for a rapid, large-scale public health response (14).

## CHALLENGES TO MENINGOCOCCAL SURVEILLANCE IN LATIN AMERICA

Countries in Latin America face several challenges in strengthening meningococcal disease surveillance. The countries need to establish more uniform, high-quality surveillance practices and use standardized case definitions. High-quality surveillance requires trained personnel at hospitals capable of collecting sterile specimens of CSF or blood. Frequently, patients may be treated with antibiotics days before meningococcal infection is suspected, making it harder to isolate bacterial agents in CSF or blood. Laboratories may not have access to technologies that permit rapid and accurate identification and analysis of the bacteria. Specimens may need to be transported long distances on poor roads to reach better-equipped laboratories, losing viability with every hour that passes during transport. Protocols for proper specimen transport must be followed to ensure likelihood of pathogen identification in the lab.

Additionally, financial resources are essential to enhancing surveillance of invasive meningococcal disease in Latin America, yet many countries in the region have limited funding to carry out surveillance efforts. Country ownership is key to ensuring that countries are committed to sustaining improved surveillance practices. In resource-constrained settings, the integration of surveillance efforts across multiple infectious diseases could provide the personnel and technology needed to meet these challenges.

## RECOMMENDATIONS TO STRENGTHEN MENINGOCOCCAL SURVEILLANCE AND DISEASE CONTROL

### Improve diagnostic capabilities

Polymerase chain reaction (PCR) is a crucial tool for improved detection of the causative agent. In 2007, Brazil incorporated real-time PCR into bacterial meningitis surveillance (15). Previously, the bacterial agent responsible for meningitis was unknown in about half of all cases in the state of São Paulo. Introduction of PCR allowed this proportion to be cut in half (15). Countries should increase efforts to ensure access to PCR and other molecular tools in hospitals likely to receive patients with meningococcal disease.

However, costly PCR equipment will unlikely become available in every hospital. Each country should assess two or three laboratory locations that can be developed to serve as central nodes for laboratory specimen analysis covering different regions of the country. Countries with the capacity for PCR-based diagnostics will also be able to utilize the equipment for the detection of other infectious diseases, such as *Bordetella pertussis*. In such instances, laboratory personnel will need to be well trained in order to ensure good laboratory practices and prevent cross-contamination of specimens.

In Argentina, the Ministry of Science, Technology and Productive Innovation is funding the development of low-cost supplies for PCR testing to avoid dependence on expensive supplies procured internationally. Other countries could undertake similar approaches to reduce costs.

When PCR is not available, filter wraps can be used to secure a specimen that is mailed to a laboratory for diagnostic testing. Ideally, analysis should take place no more than 24 hours after hospital admission, so rapid shipment is critical for this technique.

### Improve communication between epidemiologic and laboratory analyses

Communication between hospital physicians and laboratory staff needs to improve to ensure a timely flow of information to treat patients and inform public health decisions. Effective communication ensures all necessary epidemiologic information for each case is collected and matched to laboratory analysis.

Communication can be improved by having health authorities form a working alliance and call periodic meetings with physicians, epidemiologists, and laboratory personnel to exchange data.

### Improve communication between the health system and communities

Prompt and accurate communication among physicians, public health authorities, government officials, and communities is essential during an outbreak. Communication is the responsibility of providers across all levels of the health system. All people in a position to influence public understanding need to provide consistent information and messages to ensure such messages become the core story in reporting about the disease. Communication must be managed in a way that is calm, responsible, and transparent. During outbreaks, one key challenge is to manage expectations about vaccine supply and chemoprophylaxis, as meningitis quickly elicits fear among the public. Effective risk management communication requires expertise that needs to be funded and promoted within national vaccine programs.

### Improve monitoring of long-term impacts on survivors

Data on disease sequelae are not routinely collected in Latin America (16). In the region, few studies have assessed the long-term impacts of meningococcal disease on survivors. A full understanding of the impact of disease sequelae would help define the benefits of vaccination and inform cost-effectiveness studies.

Latin America would likely benefit from a regional network of hospitals to study long-term sequelae. The network could start by consolidating the highly diverse information that exists across the region. By pooling resources and data, all Latin American countries could have access to more robust information, including the serogroup-specific characteristics of sequelae, and a better understanding of potential risk factors.

Studies of sequelae typically assess hearing impairments, seizures, amputations, and skin scars. Other possible sequelae (such as arthritis, vision and renal function loss, neurocognitive disabilities, psychological or behavioral disabilities, and motor deficits) should also be considered. Current studies, such as one whose preliminary phase is now under way at the Santa Casa de São Paulo Hospital in Brazil, could serve as prototypes for similar evaluations in other places across Latin America.

### Improve knowledge of MenB vaccines

The cause of epidemics in several Latin American countries in the 1980s, serogroup B was associated with the international spread of epidemic clones of the ET5/ST32 type (10). However, developing a vaccine for serogroup B disease that provides effective protection for a long duration has been challenging.

Two new recombinant protein-based MenB vaccines have recently become available. However, it is unknown whether the vaccines provide cross-protection against other serogroups. The efficacy of MenB vaccines in different age groups, including infants, as well as the persistence of antibody titers and immunologic memory following vaccination, is not well understood. In fact, the estimated impact on carriage transmission is based on a single study (17). To date, only the United Kingdom has implemented routine vaccination against MenB disease for an entire birth cohort. The use of recombinant MenB vaccines is currently not part of routine programs in Latin America, given the limited information on effectiveness, coverage for regional serogroup B strains, duration of protection, long-term safety, and cost-effectiveness, as well as the lack of registration of the vaccines in many countries in the region.

### Improve defining and controlling epidemics

A key issue in identifying meningococcal disease outbreaks is accurately defining the population at risk. This population is generally counted within a geographic area, such as a neighborhood, district, or city. Knowing which age groups are at greatest risk is also vital.

The Centers for Disease Control and Prevention (CDC) previously defined an outbreak as a disease incidence of at least 10 cases per 100 000 people over a threemonth period (18). However, a simple calculation of whether there are 10 cases per 100 000 people is outdated and needs to be revised. The CDC is currently updating its guidelines and is likely to recommend starting vaccination earlier in outbreaks.

Meningococcal epidemiology is of concern precisely because of its epidemic potential and its propensity to give rise to hypervirulent new strains. Proper control of outbreaks can therefore have impacts on public health well beyond the immediate geographic area, and even across national borders.

## CONCLUSIONS

In Latin America, only a limited number of countries have implemented meningococcal disease surveillance programs, and the information that is collected is usually neither uniform nor easily accessible. Given the availability of several meningococcal vaccines, high-quality surveillance data is crucial when deciding the appropriateness of vaccination strategies to control the disease in the region.

Latin American countries will need to carefully decide which vaccines to use, when to use them, and in which age groups. In some contexts, experiences demonstrate that routine infant vaccination is the most effective short-term strategy to control disease in the most affected age groups. In addition, routine vaccination could be more cost-effective when such key factors as vaccine wastage, price, and uptake are mitigated.

Strengthening meningococcal disease surveillance systems will provide a more comprehensive picture of the burden of disease, as well as new insights into the epidemiology of the disease in Latin America. More specific information on evolving serogroups, the molecular profiles of disease clones, and carriage prevalence, along with analysis of disease sequelae, will all serve to advance public health.

During meningococcal outbreaks, people often ask, “How many individuals will die before taking action?” Surveillance, science, and the ethical responsibility to protect public health will rephrase the question as “How many lives can we save by taking crucial actions now?”

### Acknowledgments

The authors wish to acknowledge all the workshop participants, who were instrumental in the development of these recommendations

### Disclaimer

Authors hold sole responsibility for the views expressed in the manuscript, which may not necessarily reflect the opinion or policy of the *RPSP/PAJPH* or PAHO.
